# Surgical Practice Parameters for the Definitive Management of Sacrococcygeal Pilonidal Sinus Disease: Surgeons’ Perspective

**DOI:** 10.7759/cureus.39480

**Published:** 2023-05-25

**Authors:** Munyaradzi G Nyandoro, Mary Teoh, Andrew Thompson, David Fletcher

**Affiliations:** 1 General and Colorectal Surgery, Fiona Stanley Hospital, Perth, AUS; 2 School of Medicine, The University of Western Australia, Perth, AUS; 3 General and Colorectal Surgery, Sir Charles Gairdner Hospital, Perth, AUS; 4 General Surgery, Rockingham General Hospital, Perth, AUS; 5 General Surgery, Fiona Stanley Hospital, Perth, AUS; 6 General Surgery, Harry Perkins Institute of Medical Research, Perth, AUS

**Keywords:** flap procedure, patient factors, recurrence, self-reported practice, sacrococcygeal pilonidal sinus disease

## Abstract

Background

Sacrococcygeal pilonidal sinus disease (SPD) is a common general surgical condition encountered in practice and predominantly affects young males. Surgical practice parameters for the management of SPD are variable. This study aimed to review current surgical practice parameters for SPD management in Western Australia.

Methodology

This study conducted a de-identified 30-item multiple-response ranking, dichotomous, quantitative, and qualitative survey of self-reported surgeon practice preferences and outcomes. The survey was sent to 115 Royal Australian College of Surgeons - Western Australia general/colorectal surgical fellows. Data were analyzed using SPSS version 27 (IBM Corp., Armonk, NY, USA).

Results

The survey response rate was 66% (N = 77). The cohort comprised mostly senior collegiate (n = 50, 74.6%), and most were low-volume practitioners (n = 49, 73.1%).

For local disease control, most surgeons perform a complete wide local excision (n = 63, 94%). The preferred wound closure method was an off-midline primary closure (n = 47, 70.1%). Self-reported SPD recurrence, wound infection, and wound dehiscence rates were 10%, 10%, and 15%, respectively. The three high-ranked closure techniques were the Karydakis flap, Limberg’s flap (LF), and Z-Plasty flap. Each surgeon’s median annual SPD procedures were 10 (interquartile range = 15). The surgeons could utilize their preferred SPD closure technique (mean = 83.5%, standard deviation = ±15.6).

Univariate analysis showed significant associations between years of experience and SPD flap techniques utilized, with senior surgeons significantly less likely to use either the LF (p = 0.009) or the Bascom procedure (BP) (p = 0.034). Instead, there was a preference for using healing by secondary-intention technique (SIT) compared to younger fellows (p = 0.017). A significant negative correlation existed between practice volume and SPD flap technique utilization, with low-volume surgeons less likely to prefer the gluteal fascia-cutaneous rotational flap (p = 0.049) or the BP (p = 0.010). However, low-volume practice surgeons were significantly more likely to use SITs (p = 0.023).

The three most important patient factors in choosing SPD techniques were comorbidities, likely patient compliance, and attitude toward the disease. Meanwhile, factors influencing local conditions included the proximity of the disease to the anus, the number and location of pits and sinuses, and previous definitive SPD surgery. Key informants for technique preference were perceived low recurrence rate, familiarity, and overall good patient outcomes.

Conclusions

Surgical practice parameters for managing SPD remain highly variable. Most surgeons perform midline excision with off-midline primary closure as the gold standard. There is a clear and present need for clear, concise, and yet comprehensive guidelines on managing this chronic and often disabling condition to ensure the delivery of consistent, evidence-based care.

## Introduction

Epidemiology and etiology 

The term *pilonidal* is derived from pilus, meaning hair, and nidus, meaning nest. Sacrococcygeal pilonidal disease (SPD) is a common condition with an estimated incidence of 26 per 100,000 and was first described by Herbert Mayo in 1833 [1-5]. SPD is a disease that arises in the hair follicles of the natal cleft of the sacrococcygeal area. Its etiology involves hair (either from the head or growing in the natal cleft itself) causing a local foreign body reaction and the subsequent infection resulting in the formation of pseudocysts, abscesses, and chronic sinuses. It is seen predominantly in young adults of working age and appears in males three to four times more often than in females [1-7]. It is a chronic disabling condition that causes discomfort, which may interfere with education or work due to poor hygiene, malodor, or itching [1-9].

As most patients affected by SPD are between 15 and 40 years of age, the savings to the community in terms of working days lost would be considerable if a simple, cost-effective treatment was available [1-5]. Ideally, definitive treatment of SPD should be cost-effective, require little or no hospitalization, be associated with minimal discomfort and wound care, and have a low recurrence rate to decrease time off work or school.

Management

Many surgical techniques have been described for SPD treatment, yet a lack of consensus on the optimal surgical approach remains. Various surgical procedures for SPD have different complications, primarily recurrence and surgical site infection (SSI) to the less common wound dehiscence, maceration, hematoma, seroma, sphincter damage, and flap edema [1-11]. The American College of Colon and Rectal Surgeons’ practice guidelines suggest flap reconstruction techniques as good surgical options for chronic SPD (a strong recommendation based on moderate-quality evidence - grade lb). Still, they do not specify which flap procedure should ideally be used [12]. This is also true for the Italian and German Colorectal Surgeon’s guidelines [13,14]. Currently, there are no published Australian guidelines on SPD management.

Previous surveys

A single Australian and several contemporary European surveys have been published reflecting individual surgeon practice preferences for SPD management. The surveys show great variability in surgical practice, techniques, priorities, and perceived outcomes [15-18]. This study reviewed current surgical practice, preferences, and outcomes for elective SPD surgical management in Western Australia (WA) as part of comprehensive multicenter and patient-reported outcomes. The objective was to find the surgical technique most utilized in elective SPD management and evaluate local disease and patient factors influencing the surgeon’s practice preferences. The study also measured individual surgeons’ perceived complications, recurrence, surgical site infection, and wound breakdown rates.

## Materials and methods

Surgeon survey

The local branch of the Royal Australian College of Surgeons (RACS) was approached to provide a census of local fellows. A total of 115 general and colorectal surgeon fellows of the RACS WA branch were invited via email to participate in an online, de-identified survey of self-reported practice preferences and outcomes for elective SPD surgery. The 30-item SurveyMonkey™ containing a mix of dichotomous (yes/no), multiple responses, and free text completion items was conducted from September 2018 to March 2019 (Appendices). The survey included questions used in the prior surveys and additional questions about specific local practice preferences [15-17]. Five (monthly) reminder emails were sent during the recruitment period to improve the response rate.

As no local or published standard existed at the time of conception, this paper set a standard for SPD practice workload and dichotomized it into two categories of low and high-volume practice, with low-volume practice defined as less than 20 SPD procedures per year and likewise high-volume practice to be more than 20 per year.

SPD procedures in practice

The survey presented the respondents with a detailed description of each procedure shown, the list being Karydakis flap (KF), modified Karydakis flap (MKF), Limberg’s rotational flap (LF), modified Limberg’s rotational flap (MLF), Bascom’s cleft lift procedure (BCL), gluteus maximus myocutaneous rotational flap (GRF), Z-plasty flap (ZP), V-Y advancement flap (VY), primary open (PO), and marsupialization (MARS). Due to the low frequency of participants reporting partial primary closure, LO, and MARS, these were grouped as secondary-intention techniques (SITs).

Statistical analysis

Baseline characteristics and self-reported practice were described using mean (±standard deviation, SD), median (interquartile range, IQR), and frequencies/proportions as appropriate. Outcomes for continuous unpaired variables were analyzed with the nonparametric independent-sample Kruskal-Wallis and Mann-Whitney U tests. Univariate dichotomous results were compared between groups using the chi-square or Fisher’s exact tests with no adjustment for multiple comparisons. Influencing factors and critical informants for practice preferences were captured with the Likert scale and expressed as proportions and a 95% confidence interval (CI). Spearman’s rank correlation test assessed the correlation between two quantitative variables. Thematic analysis was done for qualitative data and expressed as logarithmic clouds. All analyses were performed using SPSS Statistics for Mac, version 27 (IBM Corp., Armonk, NY, USA), and a two-tailed p-value of <0.05 was considered statistically significant.

Permissions

Ethics approval was granted by the lead Human Research Ethics Committee (HREC) - South Metropolitan Health Service Ethics (SMHS) - RGS511 and The University of Western Australia HREC - RA/4/20/4547.

## Results

Demographics

The response rate was 66% (n = 77), of which 10 respondents indicated that they do not perform SPD surgery and were removed from further analysis. Overall, 98.5% of the respondents were General Surgeons, of whom 26.9% indicated a Colorectal sub-specialization or interest. The cohort comprised mostly senior collegiate 74.6% but had a trend toward low-volume practice at 73.1% (n = 49). There was an equal distribution of key demographics (Table 1).

**Table 1:** Demographic characteristics of the respondent surgeons. Values are median (interquartile range) and the number of participants (%) unless otherwise indicated. †: SPD = sacrococcygeal pilonidal sinus disease; ‡ Elective = definitive non-acutely infected surgery

N = 67	n	%
Specialty		
General Surgeon	66	98.5
Colorectal Surgeon/sub-interest	18	26.9
Other non-specified	1	1.5
Years of practice		
Less than 5 years	17	25.4
5 to 10 years	17	25.4
More than 10 years	33	49.3
Seniority		
Senior Consultant	50	74.6
Younger Fellow	17	25.4
SPD^†^ elective workload		
Elective^††^ procedures per year (n: median, range)	10	1–50
Low volume (less than 20)	49	73.1
High volume (more than 20)	18	26.9

Self-reported practice parameters

Most surgeons (94%) perform a complete wide local excision for local disease control, with the preferred wound closure method being an off-midline primary closure at 70.1%.

The reported practice for healing by SIT demonstrated a preference for simple wound packing over vacuum device application at 73.3% and 43.3%, respectively. More than half of the surgeons used a drain routinely, while only about a quarter used methylene blue to define diseased tissues. Most surgeons reported employing supportive measures to facilitate recovery and mitigate the risk of SPD recurrence. Shaving was the most widely adopted standard to keep the natal cleft hair free, while laser treatments were the least (Table 2). Each surgeon performed a median of 10 elective SPD procedures per year. Most could use their preferred SPD technique with a median of 90% (range = 20-100). Self-reported median SPD recurrence was 10%; other complication rates are reported in Table 3.

**Table 2:** Self-reported practice pathways and supportive measures utilization and characteristics of the respondent surgeons. Values are the number of patients (%) unless otherwise indicated. †: 95% CI = confidence interval (Wilson score interval)

N = 67		Number (n)	Proportion (%)	95% CI^†^
Primary closure pathway				
	Complete local excision	63	94.0	86–98
	Primary closure	47	70.1	58–80
	Routine drain used	34	58.6	46–70
	Methylene blue used	18	26.9	18–39
Healing by secondary intention pathway (SIT)				
	Routine wound packing	49	73.1	61–82
	Vacuum dressing	29	43.3	32–55
	Laying open/marsupialization	4	6.0	2–14
Supportive measures in practice				
	Routine shaving	50	74.6	63–84
	Dedicated clinic	34	50.7	39–62
	Waxing/loofa	30	44.8	33–57
	Depilating creams	25	37.3	27–49
	Laser hair removal	9	13.4	7–24
	Other supportive measures	5	7.5	3–16

**Table 3:** Self-reported practice utilization and complications. Values are median (interquartile range) and the number of procedures (%) unless otherwise indicated. †: SPD = sacrococcygeal pilonidal sinus disease; ‡: Not all surgeons responded; the proportion only includes valid responses.

	Median	Range
Elective SPD^† ^procedures performed per year	10	1–50
Utilization of preferred closure technique	90%	20–100
Recurrence self-reported	10%	0–30
Wound Infection ^‡^	10%	2–30
Wound Breakdown ^‡^	15%	0–50

Most surgeons reported some familiarity with common SPD procedures presented in the survey, listed in the methods, and provided in the Appendices. The surgeons were most familiar with the KF at 79.1%, while the V-Y was the least familiar at 9%. On preference ranking of SPD procedures, the surgeons chose the KF as their preferred SPD technique, followed by LF, and ZP was their third-ranked go-to technique (Table 4).

**Table 4:** Self-reported closure technique familiarity and ranked preferences of the respondent surgeons. Values are the number of respondents (%) unless otherwise indicated. † 95% CI = confidence interval (Wilson score interval)

N = 67		Number (n)	Proportion (%)	95% CI^†^
Surgeon familiarity with each closure technique				
	Karydakis flap	53	79.1	68–87
	Limberg’s flap	38	56.7	45–68
	Bascom procedure	31	46.3	35–58
	Z-Plasty flap	21	31.3	22–43
	Gluteal fascio-cutaneous rotation flap	18	26.9	18–39
	Other flap technique	17	25.4	16–37
	V-Y advancement flap	6	9.0	4–18
Surgeon ranked preferences for closure technique				
	Ranked first - Karydakis flap	35	62.5	49–74
	Ranked second- Limberg’s flap	15	34.9	22–50
	Ranked third - Z-Plasty flap	14	70.0	48–85

Influencing factors

Most surgeon’s decision-making in selecting SPD procedures for patients was highly individualized and influenced by patient factors, ranked in order of importance: (i) present comorbidities (e.g., obesity, diabetes), (ii) likely patient compliance with the treatment plan, and (iii) patient attitude toward their disease. This process was also influenced by local disease factors, ranked in descending order of importance: (a) proximity of disease to the anus, (b) number and location of primary pits and secondary sinuses, (c) and previous SPD surgery (Table 5).

**Table 5:** Self-reported ranked important influencing factors for choosing closure technique by the respondent surgeons. Values are the number of respondents (%) unless otherwise indicated. †: 95% CI = confidence interval (Wilson score interval)

N = 67		Number (n)	Proportion (%)	95% CI^†^
Important patient factors				
	Ranked first - Comorbidities	27	54.0	40–67
	Ranked second - Patient compliance	20	58.8	42–74
	Ranked third - Patient attitude	21	70.0	52–83
Important local disease factors				
	Ranked first - Proximity of disease to the anus	25	62.5	47–76
	Ranked second - Number and location of pits	24	51.1	37–65
	Ranked third - Previous SPD surgery	29	70.7	56–82

When asked to elaborate on their practice flexibility, 67.2% of the surgeons strongly agreed to use the same SPD technique for all their patients independent of the affected area. Respondents were asked to rank motivators and values in selecting particular SPD procedures, with personal preference as the main guide (82.1%). Almost half of the participating surgeons (49.3%) took into consideration patient preference in the selection process. About 79.1% of respondents cited patient factors (compliance, obesity, attitude toward the disease, diabetes) as enormously influential to their decision-making. The surgeons rated the severity or stage of local disease as the most influencing factor in selecting an SPD procedure, with 89.6% of the surgeons agreeing.

Years of experience and associations

The surgeons in the mid-tier group (5-10 years) performed significantly more SPD procedures per year, with a median of 15 (IQR = 10) procedures (p = 0.016). There were no statistical differences in the estimation rates of complications (recurrence, infection, and dehiscence) between the years of practice tiers. There were, however, significant statistical differences in the SPD technique choices being influenced by either patient preferences (p = 0.028) or by patient factors such as comorbidities (p = 0.004) between tiers of surgical experience, with more experienced surgeons (>10 years of experience) less likely to put more emphasis on these parameters (Table 6).

**Table 6:** Comparisons of continuous variables characteristics by years of practice. ‡: Nonparametric independent-sample Kruskal-Wallis test (with Benjamini-Hochberg adjusted p-values); †: Mdn = median, IQR = interquartile range, n = number; *: denotes significance at p < 0.05.

N = 67	<5 years Mdn (IQR)^ †^	5–10 years Mdn (IQR)	>10 years Mdn (IQR)	P-value^‡^
Procedures per year (n)	10 (13)	15 (10)	10 (9)	0.016*
How often able to use preferred technique (%)	85 (11)	80 (18)	90 (17)	0.216
Estimated recurrence rate (%)	15 (15)	10 (8)	10 (12)	0.522
Estimated wound infection rate (%)	10 (15)	15 (8)	10 (14)	0.608
Estimated wound dehiscence rate (%)	15 (15)	15 (10)	12 (10)	0.701
Uses the same approach all the time (n)	2 (3)	2 (3)	2 (3)	0.462
Technique is a personal preference (n)	2 (2)	2 (1)	2 (1)	0.587
Technique is a patient preference (n)	2 (3)	2 (3)	3 (2)	0.028*
Technique is influenced by local factors (n)	2 (1)	1 (1)	1 (1)	0.185
Technique is influenced by patient factors (n)	1 (1)	1 (1)	2 (2)	0.004*
Only deals with complex cases (n)	4 (2)	4 (1)	4 (2)	0.702
Technique is influenced by other factors (n)	3 (2)	3 (0)	3 (1)	0.830

Univariate analysis showed significant associations between years of experience and SPD flap techniques utilized. More senior surgeons were significantly less likely to prefer the Limberg’s flaps (p = 0.009) or the Bascom procedure (p = 0.034). Senior surgeons were more significantly associated with favoring the healing by secondary intention pathway than younger fellows (p = 0.017). Regarding supportive measures, the senior collegiate group was less inclined to set up dedicated follow-up clinics (p = 0.050) nor recommend waxing as a routine (p = 0.048).

Further analysis showed significant associations between years of experience and the factors that influenced the selection of SPD techniques. The choice of SPD procedure among the more senior surgeons was reported as less likely to be influenced by either patient preferences (p = 0.008) or patient factors (p = 0.006). Interestingly, no statistical differences existed in which group dealt with the complex cases more often. Still, there was a trend toward the more senior cohort managing such cases at 50% compared to 20% and 30% for the lower-tier groups, respectively (p = 0.888).

Practice volume

On a sub-analysis of SPD practice workload, dichotomized into low and high-volume practice as defined in methods, the high-volume group mean (procedures per year) was 25.6 (SD = ±9.7) (p < 0.001). There were no statistical differences between the two volume groups for estimated complication rates (recurrence, infection, and dehiscence). However, there were significant statistical differences in the SPD technique choices being influenced by either surgeon’s personal preferences (p = 0.029) or other specified external factors (p = 0.047) between the groups. High-volume surgeons were more likely to choose a technique based on personal preference (94.4% vs. 77.6%). Meanwhile, low-volume surgeons cited other specified factors higher in the decision-making process (Table 7).

**Table 7:** Comparisons of continuous variables characteristics by practice volume. ‡: Nonparametric independent-sample Kruskal-Wallis test (with Benjamini-Hochberg adjusted p-values) †: Mdn = median, IQR = interquartile range, n = number; *: denotes significance at p < 0.05.

N = 67	Low volume Mdn (IQR)†	High volume Mdn (IQR)†	P-value‡
Years of surgical practice (n)	3 (2)	2 (1)	0.341
Procedures per year (n)	10 (5)	20 (10)	<0.001*
How often able to use preferred technique (%)	90 (16)	85 (15)	0.136
Estimated recurrence rate (%)	10 (11)	10 (10)	0.751
Estimated wound infection rate (%)	10 (15)	10 (11)	0.791
Estimated wound dehiscence rate (%)	15 (10)	10 (11)	0.230
Uses the same approach all the time (n)	2 (3)	2 (1)	0.068
Technique is a personal preference (n)	2 (1)	1 (1)	0.029*
Technique is a patient preference (n)	3 (3)	2 (2)	0.115
Technique is influenced by local factors (n)	1 (1)	1 (1)	0.131
Technique is influenced by patient factors (n)	2 (2)	1 (1)	0.117
Only deals with complex cases (n)	4 (2)	3 (1)	0.225
Technique is influenced by other factors (n)	3 (0)	3 (2)	0.047

Associations for practice volume

Univariate analysis showed significant negative associations between practice volume and SPD flap technique utilization, with low-volume surgeons less likely to prefer the gluteal fascio-cutaneous rotational flap (p = 0.049) nor the Bascom procedure (p = 0.010). However, a significant positive association existed between low-volume practice surgeons choosing to use the healing by secondary intention pathway (p = 0.028). No statistically significant differences existed between the two volume groups for supportive measure recommendations. Still, there was a clinically significant trend for the low-volume group’s broad adoption of supportive measures (Table 8).

**Table 8:** Characteristics of practice preferences, and univariate chi-square results for independent variables, with the volume of surgical SPD practice as the dependent variable. Values are the number of respondents (%) unless otherwise indicated. *: Pearson chi-square analysis and Fisher’s exact test (for cell values <5); *: denotes significance at p < 0.05; †: SPD = sacrococcygeal pilonidal sinus disease; ‡: other specified, entered as free text, and analyzed thematically.

N = 67		Total cohort	Low volume (<20 procedures)			High volume (>20 procedures)		
		n (%)	(n)	(%)	P-value	(n)	(%)	P-value
Collegiate level								
	Senior Consultant	50 (74.6)	36	72.0	1.000	14	28.0	1.000
	Young Fellow	17 (25.4)	13	76.5		4	23.5	
Years of experience								
	Less than 5 years	17 (25.4)	13	76.5	0.099	4	23.5	0.099
	5–10 years	17 (25.4)	9	52.9		8	47.1	
	More than 10 years	33 (49.3)	27	81.8		6	18.2	
Methylene blue								
	Yes	18 (26.9)	11	61.1	0.178	7	38.9	0.178
	No	49 (73.1)	38	77.6		11	22.4	
Primary SPD† excision								
	Complete excision	63 (94.0)	46	73.0	0.105	17	27.0	0.105
	Laying open/marsupialization	3 (4.5)	3	100		0	0.0	
	Other	1 (1.5)	0	0.0		1	100	
Preferred closure technique								
	Primary closure	47 (70.1)	31	66.0	0.092	16	34.0	0.092
	Healing secondary intention	8 (11.9)	7	87.5		1	12.5	
	Combination of both techniques	12 (18.0)	11	91.7		1	8.3	
Prefers Karydakis/modified flap								
	Yes	53 (79.1)	37	69.8	0.320	16	30.2	0.320
	No	14 (20.9)	12	85.7		2	14.3	
Prefers Limberg’s/modified flap								
	Yes	38 (56.7)	25	65.8	0.121	13	34.2	0.121
	No	29 (43.3)	24	82.8		5	17.2	
Prefers gluteal fascio-cutaneous rotation flap								
	Yes	18 (26.9)	10	55.6	0.049	8	44.4	0.049
	No	49 (73.1	39	79.6		10	20.4	
Prefers Bascom procedure								
	Yes	31 (46.3)	18	58.1	0.010	13	41.9	0.010
	No	36 (53.7)	31	86.1		5	13.9	
Prefers V-Y advancement flap								
	Yes	6 (9.0)	5	83.3	1.000	1	16.7	1.000
	No	61 (91.0)	44	72.1		17	27.9	
Prefers Z-Plasty flap								
	Yes	21 (31.3)	13	61.9	0.234	8	38.1	0.234
	No	46 (68.7)	36	78.3		10	21.7	
Prefers healing by secondary intention								
	Yes	17 (25.4)	16	94.1	0.028	1	5.9	0.028
	No	50 (74.6)	33	66.0		17	34.0	
Routine use of drain								
	Yes	34 (58.6)	21	61.8	0.089	13	38.2	0.089
	No	24 (41.4)	20	83.3		4	16.7	
Routine vacuum dressing								
	Yes	29 (43.3)	24	82.8	0.121	5	17.2	0.121
	No	38 (56.7)	25	65.8		13	34.2	
Routine wound packing								
	Yes	49 (73.1)	33	67.3	0.120	16	32.7	0.120
	No	18 (26.9)	16	88.9		2	11.1	
Dedicated follow-up clinic								
	Yes	34 (50.7)	23	67.6	0.304	11	32.4	0.304
	No	33 (49.3)	26	78.8		7	21.2	
Recommends shaving								
	Yes	50 (74.6)	34	68.0	0.126	16	32.0	0.126
	No	17 (25.4)	15	88.2		2	11.8	
Recommends waxing								
	Yes	30 (44.8)	19	63.3	0.103	11	36.7	0.103
	No	37 (55.2)	30	81.1		7	18.9	
Recommends depilation creams								
	Yes	25 (37.3)	19	76.0	0.683	6	24.0	0.683
	No	42 (62.7)	30	71.4		12	28.6	
Recommends laser hair removal								
	Yes	9 (13.4)	7	77.8	1.000	2	22.2	1.000
	No	58 (86.60	42	72.4		16	27.6	
Recommend other supportive measures^‡^								
	Yes	5 (7.5)	3	60.0	0.605	2	40.0	0.605
	No	62 (92.5)	46	74.2		16	25.8	

Univariate analysis showed significant associations between the volume of practice and factors that influenced SPD techniques, with the low-volume group significantly associated with having a less flexible approach; two-thirds (64.4%) reported using the same SPD techniques all the time regardless of confounding patient or local disease factors compared to only one-third (35.6%) in the high-volume group (p = 0.038). Interestingly, there were no statistical differences between the two groups for complex cases, but there was a preponderance of low-volume surgeons managing such cases (p = 0.097) (Table 9).

**Table 9:** Characteristics of practice preferences and univariate chi-square results for independent variables, with the volume of surgical SPD practice as the dependent variable. Values are the number of respondents (%) unless otherwise indicated. *: Pearson chi-square analysis and Fisher’s exact test (for cell values <5); *: denotes significance at p < 0.05; †: SPD = sacrococcygeal pilonidal sinus disease; ‡: Other specified, entered as free text, and analyzed thematically.

N = 67		Total cohort	Low volume (<20 procedures)			High volume (>20 procedures)		
		n (%)	(n)	(%)	P-value	(n)	(%)	P-value
Uses the same SPD^† ^approach all the time								
	Yes	45 (67.2)	29	64.4	0.038	16	35.6	0.038
	No	22 (32.8)	20	90.9		2	9.1	
The technique chosen is a personal choice								
	Yes	55 (82.1)	38	69.1	0.158	17	30.9	0.158
	No	12 (17.9)	11	91.7		1	8.3	
The technique is chosen because of patient preferences								
	Yes	33 (49.3)	21	63.6	0.084	12	36.4	0.084
	No	34 (50.7)	28	82.4		6	17.6	
The technique is chosen because of patient factors								
	Yes	53 (79.1)	37	69.8	0.320	16	30.2	0.320
	No	14 (20.9)	12	85.7		2	14.3	
The technique is chosen because of local disease factors								
	Yes	60 (89.6)	42	70.0	0.176	18	30.0	0.176
	No	7 (10.4)	7	100		0	0.0	
The technique is chosen because only deals with complex disease								
	Yes	10 (14.9)	9	90.0	0.267	1	10.0	0.267
	No	57 (85.10	40	70.2		17	29.8	
The technique is chosen because of other specified factors^§^								
		8 (11.9)	8	100	0.097	0	0.0	0.097
		59 (88.1)	41	69.5		18	30.5	

Thematic analysis

**Figure 1:**
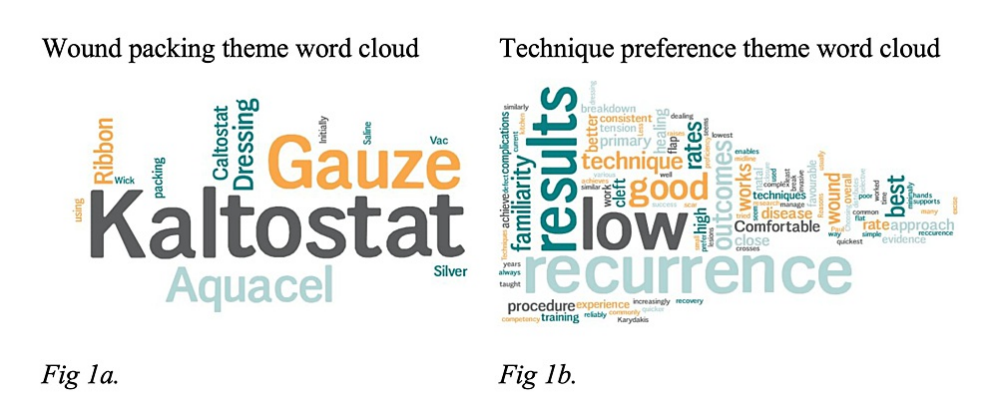
Wound packing and technique preference theme word cloud.

For the healing by secondary intention pathway, when opting to pack the wound, the most cited preferential packing material was Kaltostat (62.2%), ribbon gauze (15.6%), and Aquacel (13.3%). The logarithmic thematic pictorial representation is demonstrated in Figure 1a. The key informants for SPD technique preference were low recurrence rates, familiarity, and overall good outcomes (Figure 1b).

## Discussion

Practice parameters

SPD is a common general surgical condition encountered in practice, predominantly in the young adult male working population [1-7]. The ideal definitive treatment for SPD should be one that requires little to no hospitalization, is associated with minor discomfort, has low recurrence rates to minimize time off from work or school, and has a low burden on the healthcare system [1-5]. Multiple surgical techniques have been developed, adapted, and modified to address this vexatious disease. Nevertheless, a lack of consensus on the optimal SPD technique persists due to the challenging nature of its management, with no one approach better than another [1-7]. This study’s results confirm that SPD surgical practice parameters remain variable in utilization and application. Many participants reported using more than one flap technique, with 18% also reporting using a combination of primary flap closure and SIT techniques.

Practice preferences

Consistent with the published literature, in this study, most surgeons still prefer to perform a complete wide local excision (94%) for local disease control, with a preference for an off-midline primary closure (70%), deemed best practice, as recommended by the current American [12] and European Guidelines [13,14]. More than half of the surgeons used a drain as a routine. The routine use of surgical drains in conjunction with flap techniques has demonstrated a decrease in recurrence rates or surgical site infections; however, it is associated with decreased incidence of flap edema. Therefore, the American practice guidelines [12] advocate for the individualized use of surgical suction drains, a strong recommendation based on moderate-quality evidence (grade lB).

Methylene blue

About a quarter of the respondents in this study reported using methylene blue for tissue definition. However, its use remains controversial because it can be associated with significant staining of normal tissue resulting in unnecessarily extensive excisions. Additionally, various studies have not demonstrated a substantial reduction in recurrence rates with the additional staining step. Moreover, the second part of this study (retrospective decade review of all local elective SPD outcomes) interestingly showed that methylene blue use was significantly and independently associated with achieving clear surgical margins on histopathology, which were then subsequently associated with low rates of recurrence (p < 0.001) [19].

Breadth of practice

This study captured current practice parameters from a diverse pool of experienced surgeons and showed that most responding participants were senior collegiate (74.6%). Consistent with published practice volume literature [15-18], this study showed a predominantly low volume of local SPD practice (73.1%). No statistical differences were reported between the low-volume and high-volume groups’ self-reported recurrence rates or other key complication rates. Additionally, low-volume surgeons were reported to perform a more significant proportion of complex cases. It is possible that the low-volume practice was established to serve uncomplicated cases, thus freeing up more time and operative space for more complex and time-consuming cases to be done selectively to reduce the risk of surgeon fatigue and poor patient outcomes; however, this does not appear to be the case. Previous studies have argued that SPD practice should be concentrated in high-volume centers [15-17]. However, this survey highlights that many complex cases were performed at the low-volume center, with no significant differences in reported outcomes. Therefore, it may seem counterintuitive for SPD procedures to reallocate cases based on the argument of low-volume practitioners. This may be an area of further investigation as this study was not designed to explore this subject matter but provides insight into potential future studies.

Practice flexibility

It is well-known that there is no one-size-fits-all approach in SPD management. This study identified that more than two-thirds of the surveyed surgeons reported using the same SPD technique for all their patients regardless of the circumstances; this is not ideal as it fails to consider each patient’s conditions, such as the severity of disease and natal anatomy, which are factors known to influence outcomes. The low-volume group was significantly more flexible in the SPD approach and application. The modest adoption of SIT reported in this study is a concern because SIT is well documented in the literature to be associated with significant human factor costs (prolonged time to heal and pain) and is resource-intensive to the healthcare system. Moreover, SPD mainly affects young patients, who make up the bulk of the workforce; thus, performing SIT procedures on them, which are known to have prolonged healing and translate into lost productive economic time, should be avoided. Therefore, it can be argued that emphasis should be given to technique selection and consideration of patient factors to ensure minimal disruption to the patient’s daily life and productive economic participation while attaining a low recurrence rate.

Supportive measures

Supportive measures such as dedicated follow-up clinics or hair removal recommendations after definitive SPD procedures remain inconsistent. In this study, the surgeons’ self-reported practice of implementing supportive measures received a mixed response inversely associated with surgical experience. The senior collegiate group was less inclined to set up follow-up clinics or recommend routine waxing. The analysis of utilizing these measures based on volume practice showed no statistical significance. However, there was a noticeable trend for the low-volume group’s broader implementation of these measures. A recent systematic review by Pronk [20] showed the benefit of implementing supportive measures for risk reduction; however, due to the limitations of the review, a recommendation was made for further high-quality RCT studies. It is reasonable to adopt these secondary preventative measures post-definitive surgery as these simple measures are not inferior to the option of doing nothing [21].

Practice guidelines

The three parts of this study reflect the vast variability in SPD treatment and management in the Australian cohort [19,21]. The mixed responses from the surgeons’ self-reported practice showed different levels of familiarity with SPD techniques, differing routines regarding flexibility in technique selection based on key informants, and supportive measurements. The American [12], Italian [13], and German [14] surgical societies have published guidelines to standardize the management of a seemingly simple disease that can unfortunately quickly become a persistent burden. It is perhaps time for Australians to develop and establish a national guideline built on the foundation of international data and further adapted with local data to reflect and better serve the Australian patient population.

Study limitations

There are certain limitations and strengths of this study. This study’s comprehensive and circular/resampling nature is a strength that gives it internal validity by capturing similar responses at different stages of the survey. This is further augmented by the high response rate from an anonymous random sample of surgeons. There remains a small risk of non-response bias, but the high response rate should mitigate significant influence on results. One of the limitations of this study is the inclusion of self-reported clinical practice, as it would be impractical to verify and assess each respondent’s current practice. However, numerous studies have defended its continued use because of its high sensitivity and moderate specificity, especially in health professionals’ studies [22]. While this study acknowledges that limited value is placed on survey questionnaires in terms of drawing definitive conclusions, there is an undisputed value that this survey adds to the growing body of evidence in highlighting informative key and essential variations in current SPD practice. An outcome congruent with findings in other reported studies, this, in turn, gives this study external validity.

Future direction

A comprehensive guideline by the Colorectal Surgical Society of Australia and New Zealand would help bridge the knowledge gap in managing this debilitating disease, which primarily afflicts a young population and active economic participation.

## Conclusions

Surgical practice parameters for managing SPD remain highly variable. Most surgeons perform midline excision with off-midline primary closure as the gold standard. There is a clear and present need for clear, concise, and yet comprehensive guidelines on managing this chronic and often disabling condition to ensure the delivery of consistent, evidence-based care.

This paper is the opening salvo and is followed by a compressive medical records review (inpatient and outpatient) to capture the patient journey before closing the loop with a novel and unprecedented prospective follow-up of SPD patient-reported outcomes to truly capture the entire patient journey that can inform further practice.

## Appendices

**Table 10:** Supplemental online materials - pipetted survey questionnaire.

Supplemental online materials - pipetted survey questionnaire	
Page one	Project Title: Pilonidal Sinus Disease: Western Australia - Definitive Surgical Practice Preferences, Outcomes
	Q1. I hereby agree to be a participant in the above-named research. YES or NO
Page two	Q2. Do you perform Elective Pilonidal Sinus Surgery? YES or NO
	Q3. Are you are General Surgeon? YES or NO
	Q4. Are you a Colorectal Surgeon? YES or NO
	Q5. How many years have you been practicing as a consultant surgeon? Less than 5 years OR 5–10 years OR >10 years
Page three	Q6. How many definitive elective pilonidal sinus operations do you perform per year? Enter number
	Q7. Do you routinely use Methylene Blue Dye? YES or NO
	Q8. What is your most common elective management of the involved tissue? Complete local excision OR Laying open/marsupialization OR Minimally invasive/laparoscopic approach OR Other (please specify)
Page four	Complete excision pathway
	Q9. After wide local excision of the sinus tracts and pits, what is your most preferred technique for closure? Primary closure OR Healing by secondary intention OR Both If PRIMARY closure, then the respondent will be directed to the following questions
	10. Which of the following techniques have you used for Primary Closure in elective cases? · Karydakis Procedure/Modified (Involves a lateral elliptical excision around the disease. The procedure results in a wound that is lateral to the midline, with a shallower natal cleft) · Limberg Rotational Rhomboid Flap/Modified (It is basically a parallelogram with two angles of 120° and two of 60°, All sides of the rhomboid and all sides of the flap are equal. The flap design places the longitudinal axis of the rhomboid excision parallel to the line of minimal skin tension) · Gluteal Rotation Flap (Tissue is rotated into the defect. The procedure involving the classic rotation flap can be thought of as the closure of a triangular defect. The defect can be visualized as a portion of a much larger circle. By cutting along the arc of the circle, tissue is freed to rotate into the defect) · Bascom Procedure (Cleft Lift) (The procedure involves the removal of scarred or pitted midline skin and skin from one side of the natal cleft. The skin on the opposite side of the cleft is mobilized past the edge of the natal cleft on the other side. The skin flap is then closed over the “shallowed” valley and sutured to the side outside the cleft. · V-Y Advancement Flap (a V-Y advancement flap is created by making a V-shaped incision and advancing the broad base of the V into the defect. The resulting defect is closed primarily in a Y-shape) · Z-Plasty Flap (The transposition of two triangular flaps. The incisions are designed to create a Z shape with the central limb aligned with the part of the scar that needs lengthening or re-aligning) · Other (please specify) – Please specify
	Q11. Of the techniques selected above (Q.10), Which are your THREE most preferred techniques for managing Pilonidal Sinus Disease in elective cases? Karydakis Procedure/Modified, OR Limberg Rhomboid Rotational Flap/Modified, OR Gluteal Rotation Flap, OR Bascom Procedure (Cleft Lift), OR V-Y Advancement Flap, OR Z-Plasty Flap OR) OR Other as specified in Q.10
	Q12. Do you routinely place a DRAIN post definitive elective pilonidal sinus surgery? YES OR NO
Page five	Complete excision pathway If HEALING by secondary intention, then the respondent will be directed to the following questions
	Q13. If healing by secondary intention, do you routinely make use of a Vacuum dressing? YES OR NO
	Q14. If healing by secondary intention, do you routinely pack the wound? YES OR NO
	Q15. If yes, which packing material do you use? Enter Choice
Page six	Q16. How often are you able to use your preferred technique? Number as (0–100) %
	Q17. In your practice using your preferred technique, what do you estimate is your recurrence rate for pilonidal sinus disease? Number as (0–100) %
	Q18. In your practice using your preferred technique, what do you estimate is your wound infection rate for pilonidal sinus disease? Number as (0–100) %
	Q19. In your practice using your preferred technique, what do you estimate is your wound breakdown rate for pilonidal sinus disease? Number as (0–100) %
	Q20. What other supportive measures do you employ? Dedicated clinic/Nurse OR OR Shaving OR Waxing/Loofra OR Depilating agents OR Laser hair removal OR Other (please specify)
Page seven	21. Please RANK the THREE most important PATIENT FACTORS in choosing operative technique (Elective Cases) Gender, Co-morbidities (Obesity, Diabetes), Age, Employment status, Attitude towards disease by the patient, Likely patient compliance, Patients concern (cosmesis), Other (please specify)
	22. Please RANK the THREE most influential LOCAL FACTORS in choosing the operative technique (Elective Cases) Recurrence rates OR Hirsutism OR Deepness of the gluteal cleft, OR Proximity of disease to anus OR Number and location of primary and secondary pits, OR Previous surgery for disease OR All of the above, OR Other (please specify)
	23. Why do you use the technique that you use for Elective cases? Free text space box option
Page eight	How strongly do you Agree or Disagree with each of the following statements about the technique/s you use for Elective cases? On a scale of 1. Strongly Agree, 2. Agree, 3. Neutral, 4. Disagree, 5. Strongly Disagree
	Q24. I use the same approach for all my patients, independent of the affected area
	Q25. The technique is chosen because it is a Personal preference
	Q26. The technique is chosen because it is a Patient preference
	Q27. The technique is influenced by Patient factors e.g. (Compliance, obesity, attitude towards the disease, diabetes)
	Q28. The technique is influenced by Local factors e.g. (Severity/Stage of disease)
	Q29. The technique is chosen because I ONLY deal with complex cases
	Q30. Other as previously specified in Q23
Page nine	31. I would like to receive an executive summary of the research findings Yes or No
	32. If you answered YES to the above question, please provide your preferred method for correspondence RACS email circulars Other (please specify)
	Please note that this research team takes your confidentiality very seriously. At no time will the raw data collected in this survey be released to any third party. End of the survey.
	https://www.surveymonkey.com/r/PilonidalSurgeryWA

## References

[1] Al-Khamis A, McCallum I, King PM, Bruce J (2010). Healing by primary versus secondary intention after surgical treatment for pilonidal sinus. Cochrane Database Syst Rev.

[2] Hodges RM (1880). Pilo-nidal sinus. Boston Med Surg J.

[3] Hull TL, Wu J (2002). Pilonidal disease. Surg Clin North Am.

[4] Patey DH, Scarff RW (1946). Pathology of postanal pilonidal sinus; its bearing on treatment. Lancet.

[5] Karydakis GE (1992). Easy and successful treatment of pilonidal sinus after explanation of its causative process. Aust N Z J Surg.

[6] Sit M, Aktas G, Yilmaz EE (2013). Comparison of the three surgical flap techniques in pilonidal sinus surgery. Am Surg.

[7] Kapan M, Kapan S, Pekmezci S, Durgun V (2002). Sacrococcygeal pilonidal sinus disease with Limberg flap repair. Tech Coloproctol.

[8] Horwood J, Hanratty D, Chandran P, Billings P (2012). Primary closure or rhomboid excision and Limberg flap for the management of primary sacrococcygeal pilonidal disease? A meta-analysis of randomized controlled trials. Colorectal Dis.

[9] Enriquez-Navascues JM, Emparanza JI, Alkorta M, Placer C (2014). Meta-analysis of randomized controlled trials comparing different techniques with primary closure for chronic pilonidal sinus. Tech Coloproctol.

[10] Aydede H, Erhan Y, Sakarya A, Kumkumoglu Y (2001). Comparison of three methods in surgical treatment of pilonidal disease. ANZ J Surg.

[11] Petersen S, Koch R, Stelzner S, Wendlandt TP, Ludwig K (2002). Primary closure techniques in chronic pilonidal sinus: a survey of the results of different surgical approaches. Dis Colon Rectum.

[12] Steele SR, Perry WB, Mills S, Buie WD (2013). Practice parameters for the management of pilonidal disease. Dis Colon Rectum.

[13] Segre D, Pozzo M, Perinotti R, Roche B (2015). The treatment of pilonidal disease: guidelines of the Italian Society of Colorectal Surgery (SICCR). Tech Coloproctol.

[14] Iesalnieks I, Ommer A, Petersen S, Doll D, Herold A (2016). German national guideline on the management of pilonidal disease. Langenbecks Arch Surg.

[15] Burnett D, Smith SR, Young CJ (2018). The surgical management of pilonidal disease is uncertain because of high recurrence rates. Cureus.

[16] Shabbir J, Chaudhary BN, Britton DC (2011). Management of sacrococcygeal pilonidal sinus disease: a snapshot of current practice. Int J Colorectal Dis.

[17] Wysocki AP (2015). Defining the learning curve for the modified Karydakis flap. Tech Coloproctol.

[18] Fabricius R, Petersen LW, Bertelsen CA (2010). Treatment of pilonidal sinuses in Denmark is not optimal. Dan Med Bull.

[19] Nyandoro M, Thompson A, Teoh M, Fletcher D (2019). Pilonidal sinus disease (PNS): a nine-year multi-centre experience and patient perspective. ANZ J Surg.

[20] Pronk AA, Eppink L, Smakman N, Furnee EJ (2018). The effect of hair removal after surgery for sacrococcygeal pilonidal sinus disease: a systematic review of the literature. Tech Coloproctol.

[21] Nyandoro M, Teoh M, Thompson A, Fletcher D (2022). Sacrococcygeal pilonidal sinus disease: patient-reported outcomes, a 10-year follow-up. Ann Coloproctol.

[22] Nyandoro MG, Kelly DA, Macey DJ, Mak DB (2016). Student-centered interventions the key to student health care worker influenza vaccination. Infect Dis Health.

